# Quantitative Detection of *Leishmania* in *Rhipicephalus microplus* and *Amblyomma sabanerae* in the Peruvian Amazon Basin

**DOI:** 10.3390/tropicalmed7110358

**Published:** 2022-11-07

**Authors:** Jesús Rojas-Jaimes, Germán H. Correa-Núñez, Lisa Donayre, Andres G. Lescano

**Affiliations:** 1 Facultad de Ciencias de la Salud, Universidad Privada del Norte, Lima 15306, Peru; 2 Escuela de Medicina Humana, Universidad Científica del Sur, Lima 15307, Peru; 3 Departamento Académico de Ciencias Básicas, Universidad Nacional Amazónica de Madre de Dios, Puerto Maldonado 17001, Peru; 4 Emerge, Emerging Diseases and Climate Change Research Unit, School of Public Health and Administration, Universidad Peruana Cayetano Heredia, Lima 15102, Peru

**Keywords:** quantitative PCR, *Leishmania*, tick, host, reservoir

## Abstract

Leishmaniasis is a disease of public importance with a complex transmission cycle. A quantitative PCR was developed by using the small subunit of the ribosomal RNA gene (SSU rRNA) as a DNA target, which is conserved in all *Leishmania* species. A TaqMan ^®^ probe was designed to have a high specificity. In all, 22 out of 23 (95.7%) ticks classified as *R. microplus* tested positive for *Leishmania* sp. The quantification was between 34.1 and 2197.1 parasites per tick in a range of 12 to 769 fg/uL. In addition, 9 out of 10 (90%) ticks classified as *Amblyomma sabanerae* tested positive for *Leishmania* sp. The quantification was between 448.6 and 5428.6 parasites per tick in a range of 157 to 1900 fg/µL. *Leishmania* sp. was identified in very high percentages in *Rhipicephalus microplus* and *Amblyomma sabanerae* from wild *Pecari tajacu* and *Chelonoidis denticulata*, in quantities of 34.1 and 5428.6 parasites per arthropod, and this could suggest that the ticks were parasitized by sucking blood from the animals from which they were collected. This is the first report about *Leishmania* parasites found in wild *Rhipicephalus microplus* and *Amblyomma sabanerae*, adding new information about the distribution and epidemiology of the parasite in sylvatic areas.

## 1. Introduction

Leishmaniasis is a disease of public importance with a complex transmission cycle. Control measures are complicated due to the participation of several factors in the spread of the disease, including the different species of the vector, the numerous reservoirs and/or hosts (among them the human being), and the climatic and social conditions [1]. Approximately 12 million people are infected worldwide, and there are an estimated 0.9 to 1.6 million new cases annually, corresponding to 55,000 cases of cutaneous and mucosal leishmaniasis and 3500 cases of visceral leishmaniasis per year in America [2]. This disease affects at least 98 countries, with an emphasis on rural areas [3]. It has been reported that there are at least 30 different species of *Leishmania*, and only one group of them (comprising 15 species), belonging to the subgenus *Viannia*, is pathogenic to humans [3,4].

Polymerase Chain Reaction (PCR) is a useful molecular tool for the detection of *Leishmania* parasites, and it can reach a sensitivity of 100% depending on the molecular marker that is used [5]. There are different molecular markers used to detect and identify the species of the genus *Leishmania* located in the nuclear or mitochondrial DNA at the level of the maxicircle or minicircle [4]. Each marker has certain advantages over the other, such as stability, number of copies, and polymorphisms [4]. The kinetoplast DNA of the parasite is one of the most used markers to detect *Leishmania.* Therefore, the design of the primers and the verification of homologous and/or complementary sequences by using bioinformatics and the size of the amplified product are important to avoid false positives [6].

Recently, the role of ectoparasites such as ticks, fleas, and lice has been attracting increasing interest in the arthropods present in the areas of visceral leishmaniasis (in some cases, in the absence of the natural vector *Lutzomyia*) and their role as effective transmitters of pathogens. Several studies have reported the presence of *Leishmania* DNA in ticks by molecular methods, which is why the involvement of these arthropods in the cycle of transmission of the causative agent of leishmaniasis is presumed [7,8].

Ticks are potential transmitters of several pathogens due to their wide distribution, high reproduction rates, blood-suction capabilities, and longevity [8,9]. Studies in France and Brazil have shown the viability of *Leishmania* and the development of this hemoparasite in the digestive tract of ticks [8,10].

Ticks can belong to two large families: *Ixodidae* (hard ticks) and *Argasidae* (soft ticks). The genus *Rhipicephalus* is found within the family *Ixodidae* and can transmit a series of pathogens that cause conditions such as Lyme disease, rickettsiosis, and encephalitis [11].

Several pathogens of veterinary and zoonotic importance, such as *Ehrlichia canis*, *Hepatozoon canis*, *Rickettsia* sp. and *Leishmania* (*Leishmania*) *infantum*, have been found in *R. sanguineus*, using molecular biology diagnostics [12]. In a study in Brazil, 10.1% of *R. sanguineus* collected from symptomatic and asymptomatic dogs tested positive for *Leishmania* (*L.*) *infantum* in 12.2% of dogs collected from an endemic area of canine leishmaniasis [9,13]. One study reported an interesting observation of *Leishmania infantum* in another species within *Ixodidae* ticks collected from dogs and a cat [14]. In addition to identification, it is extremely important to quantify the parasitic load of *Leishmania* before, during, and after the treatment of the disease since this quantification can become important in epidemiological studies and in the development of vaccines [15]. It is important to mention that the incrimination of an arthropod as a vector requires demonstrating that the parasite is capable of replicating and maturing toward an infective stage such as the metacyclic promastigote in the case of *Leishmania*, for which other complementary studies are necessary to determine the role of ticks as vectors of the parasite.

The goal of the present study was to identify and quantify the parasitic load of *Leishmania* in *R. microplus* and *A. sabanerae* collected from wild *Pecari tajacu* and *Chelonoidis denticulata*, evaluating the percentage of positive ticks for *Leishmania* sp. Ticks collected from *P. tajacu* and *C. denticulata* were studied because these animals are frequently hunted for their meat in endemic areas of leishmaniasis in the Peruvian Amazon, where hunters are infested by ticks and report having developed cutaneous leishmaniasis after being bitten by the arthropods.

## 2. Materials and Methods

### 2.1. Ethical Aspects and Fieldwork

Our research was approved by the Office of Public Health and Environment of the Regional Council of Madre de Dios, Peru. In this investigation, the tests for the identification of *Leishmania* sp. were completed with laboratory procedures in accordance with international guidelines on animal research and in agreement with the institutional Animal Care and Use Committee. The collection of ticks from wild animals was carried out in endemic zones of leishmaniasis, where people are constantly bitten by the arthropods when they perform agriculture, wood extraction, and hunting.

### 2.2. Collection and Taxonomic Classification

A total of 23 ticks (12 males, 7 females, and 4 nymphs) were collected from one *Pecari tajacu* (“collared peccary”) in the area of Botijón, a village (12°07′12.95″ S, 69°04′31.47″ W; WGS 84 and 267 m above sea level) near Planchón town, the capital of Las Piedras district. Similarly, 10 male ticks were collected from one *Chelonoidis denticulata* “motelo” from the Gamitana Farm, Las Piedras district, located on the left bank of the Bajo Madre de Dios River (12°30′36.76″ S, 68°58′49.3″ W; WGS 84 and 250 m above sea level). These collections were made during the daytime, between 9:00 a.m. and 11:00 a.m., as *P. tajacu* and *C. denticulata* are diurnal.

Both places where the ticks were collected are located in the Tambopata province, southeast of the Madre de Dios department in Peru, belonging to the humid–subtropical climate with a variable annual average temperature between 25 and 42 °C, and rainfall from 1000 to 2000 m^3^.

A total of 33 ticks were collected and stored individually in ethyl alcohol (96%) for preservation until they were transported to the Entomology Laboratory of the National Institute of Health “INS, Lima, Peru” and the Microbiology Laboratory at Universidad Científica del Sur, Lima, Peru, for identification, following the protocol published by Barros-Battesti [16].

### 2.3. Molecular Study

Collected ticks were taken to the laboratory and sectioned on a surface cleaned with absolute ethanol. Subsequently, the soft tissue was removed by using a new scalpel for each tick. After sectioning each tick, the surface was cleaned with 5% bleach. DNA extraction was performed by using ^©^Roche’s High Pure PCR Template Preparation kit (Roche Diagnostics GmbH, Roche Applied Science, 68298 Mannheim, Germany), followed by the addition of 200 µL of elution buffer [17].

The positive controls used were 5ASK (*L. major*) and MHOM/PE/84/LC53 (*L. braziliensis*), molecular-biology-grade water was used as a negative control, and the qPCR was performed under the following conditions: The target DNA for amplification was the conserved small subunit of the ribosomal RNA gene (SSU rRNA) consisting of 160 copies in each parasite cell and a DNA equivalent of 70 fg per cell [18,19]. The flanking primers used were LEIS.U1 (5′-AAGTGCTTTCCCATCGCAACT-3′) and LEIS.L1 (5′-GACGCACTAAACCCCCTCCAA-3′); additionally, a specific probe was designed to amplify a 67 bp fragment, using the LEIS.P1 fluorochrome (FAM 5′-CGGTTCGGTGTGTGGCGCC-3′ TAMRA), TaqMan ^®^ probe. The molecular protocol in the qPCR was based on a previous study [19]. The Run On Software Version was Rotor-Gene Q Software 2.3.1.49-Machine Serial No. 1013165.

To correctly calibrate the quantitative molecular patterns, a standard curve performance was developed, and the detection threshold was determined.

### 2.4. Quantitative Analysis

The final number of parasites per tick was obtained by the following equation:

Parasite/tick = fg/µL (detected by qPCR in a sample) × 200 (elution factor of DNA)/70 (factor in nanograms equivalent to a parasite). The concentration of 70 fg is equivalent to 1 *Leishmania* parasite, according to our previous validation study [19].

## 3. Results

The ticks were collected from the animals located in the jungle. Interestingly, all ticks collected from *C. denticulata* were stuck in its shell, and all ticks from *P. tajacu* were found in the skin of the abdominal area. This implies that ticks are adapted to suck blood through soft surfaces, such as skin, as well as hard surfaces, such as the shell of turtles.

**Figure 1:**
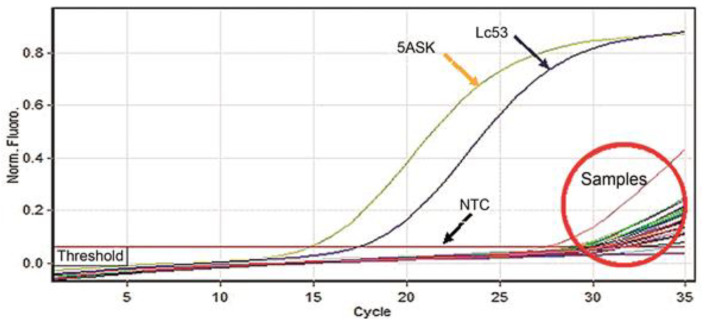
Threshold and cycle of the amplifications of the controls (5ASK-*L. major*), (MHOM/PE/84/LC53-*L. braziliensis*), and NTC (no template control) and samples.

**Figure 2:**
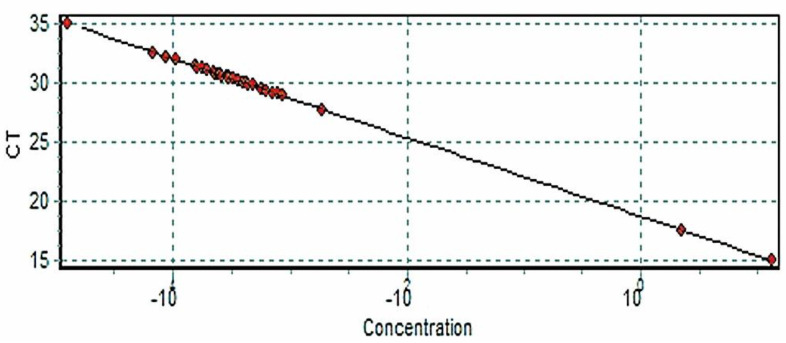
The standard curve shows the amplification (CT) on the ordinate axis, “Y”, and the known DNA in ng/µL concentration in a series of serial dilutions on the abscissa axis, “X”.

The standardization values from the process of quantification and cycling were developed correctly. The concentrations of *Leishmania* DNA in the samples were lower. compared to the controls (Figure 1 and Figure 2).

Taxonomic identifications resulted in a specific genus and species: all *R. microplus* for *P. tajacu*, and all *A. sabanerae* for *C. denticulate*. Very high percentage rates of parasitized ticks were observed (Table 1).

## 4. Discussion

The study quantified the parasite’s deoxyribonucleic acid (DNA) in *R. microplus* and *A. sabanerae*. The DNA’s values in *R. microplus* were between 12 and 769 fg/µL and between 34.1 and 2197.1 parasites per tick. In the case of *A. sabanerae*, the DNA values were between 157 and 1900 fg/µL and between 448.6 and 5428.6 parasites per tick. However, lower values of DNA have been found in another study on *R. sanguineus* [20], so it was interesting to find higher values of parasitized ticks. The quantitative PCR (qPCR) used in our study was based on the amplification of the small subunit of the ribosomal gene for ribonucleic acid (RNA) small subunit ribosomal ribonucleic acid (SSU rRNA), which consists of 160 copies present for each cell of the parasite previously validated in the Brazilian study [20].

Although *Leishmania* kinetoplast DNA (kDNA) could also be used for parasitic quantification since it has a stable number of 10,000 copies per cell [21] and previous studies have already reported the advantage of using the kinetoplast minicircle to identify the genus of the parasite *Leishmania* because of the high number of copies present at the mitochondrial level (although it is not used as a gold standard test) [4], we instead highlight the advantages of using qPCR, using SSUrRNA, due to marker stability and the sensitivity and specificity of the test [19]. Additionally, the TaqMan probe used is highly specific for *Leishmania* sp. and guarantees the reliability of the parasite detection [22].

Overall, qPCR is very sensitive and useful in evaluating the parasitic load during treatment against *Leishmania*. In an Italian study, the average number of minicircles in the kDNA was determined to be 26,556 through the usage of a qPCR and a High-Resolution Fusion PCR (HRM) to quantify and determine the differences between the subgenus *Leishmania* and *Viannia* [3]. In Brazil, a molecular study showed that 42.74% of a group of parasites composed of ticks, fleas, and lice tested positive for *L.* (*V*). *braziliensis*, 42.67% of the parasites tested positive for *R. sanguineus,* and the average amount of parasitic load found was 14.14 fg/uL, with a range between 0.37 and 113.37 fg (Ct 38.60–Ct 31.38 respectively), with the average being greater than the quantity of DNA found in *Leishmania* (*L.*) *infantum*, which was 9.10 fg/uL, as reported in the study [20]. The same study used a conventional PCR and qPCR to detect the parasitic genome, resulting in many negative tests by the conventional PCR, despite amplification by the qPCR [20]. This is probably due to the low parasitic load in the samples only testing positive through the usage of a more sensitive molecular method such as qPCR [20]. Other research carried out in France designed a quantitative real-time PCR by using the *L. major* kDNA to evaluate the parasitic load in animal tissues, detecting up to 83.3 fg/µL of DNA per reaction, equivalent to 0.08 parasite/µL, although its sensitivity was not better than compared to other studies where detection could be determined at values below 83.3 fg/µL [15].

In regard to the parasitic load, it is important to highlight that, according to previous studies, the natural vector of *Leishmania* (*L.*) *infantum* that transmits the parasites that cause visceral leishmaniasis, *Lutzomyia longipalpis* is found in its parasitized form at low percentages: 0.28% in Venezuela, from 0.29% to 0.9% in Colombia, and from 0.2% to 0.5% in Brazil, demonstrating the low probability in natural form of transmission of the parasite with respect to the number of infected arthropods [8].

A Brazilian study was able to infect hamsters with *R. sanguineus* collected from dogs with visceral leishmaniasis. The ticks were crushed into pools and were inoculated intraperitoneally and orally to the hamsters. Of these hamsters, 41.2% were infected, with 85.7% infected intraperitoneally and 14.3% infected orally, thus demonstrating the parasite’s viability in ticks and its infectivity [8]. However, the role of ticks as vectors of the parasites causing leishmaniasis has not yet been demonstrated. In the arthropod, it is expected to find the promastigote form of the parasite. This phase of the parasite is flagellated and extracellular and needs to be identified by microscopy dependent on the parasitemia of the vector, the sensitivity, and specificity of the microscopic technique. This form has been reported only in a study where it was located in the intestines, salivary glands, and ovaries of *R. sanguineus*, according to histopathological studies and corroborated by Real-Time PCR Detection [23]. Therefore, ticks and other different diptera from *Lutzomyia* sp., such as *Tabanus importunus*, fir which *Leishmania* sp. were detected, must be studied as probable mechanical or natural vectors [7,18].

Additionally, another study detected the presence of *Leishmania* (*L.*) *infantum* kinetoplast DNA from the intestines and salivary glands of *R. sanguineus*, in addition to isolating promastigotes in culture media, determining the viability of *Leishmania* [24]. Other studies have been able to identify the molecular presence of *Leishmania* in ticks and/or their hosts, showing the importance of searching for the parasite in arthropods different from the natural vector that are *Lutzomyia* and *Phlebotomus* [25,26,27,28].

In our study, most of the *R. microplus* ticks that tested positive for *Leishmania* were male (52.2%); this may be due to the male’s need to look for a partner and parasitize several hosts in search of food, according to a previous study where *Leishmania* (*L.*) *infantum* was found in 92.3% of males of *R. sanguineus* compared to 7.7% of females [13]. However, a study in Iran showed that, in the genus of *R. sanguineus,* the female ticks were the most parasitized, thus highlighting the possibility of transovaric transmission of the hemoparasites to the offspring [10].

In our study, the hematophagous role of *Rhipicephalus* (*Boophilus*) *microplus* for *Pecari tajacu* was observed. In the case of *A. sabanerae*, in which all the specimens were adult male, the hematophagous role of this tick was observed for *Chelonoidis denticulata*.

It is very important to note that 95.7% of *R. microplus* ticks collected from *P. tajacu* tested positive for *Leishmania*, highlighting the importance of new studies to elucidate the probability of *R. microplus* as a vector of *Leishmania* sp. and *Pecari tajacu* as a probable reservoir/host. Our hypothesis is based on a previous study in which *L. guyanensis* was detected in *R. microplus* collected from *Pecari tajacu*. Considering this premise, the present study was developed with the intention of investigating the percentage of parasitized ticks and the amount of *Leishmania* in *R. microplus* collected from *Pecari tajacu*; only 7% of them were detected to be positive in the previous study [29]. Another interesting finding was that 90% of males of *A. sabanerae* tested positive for *Leishmania*. Therefore, there is a high percentage of parasitized ticks in this study in the Peruvian jungle.

The genus *Amblyomma* is related to transmissions of various pathogens. For example, an interesting study in Madre de Dios, Peru, found *Bartonella bacilliformis* in *Amblyomma scalpturatum* and *Amblyomma ovale* collected from *Tapirus terrestris* for the first time. *Bartonella bacilliformis* is the causal agent of “Carrion Disease”, a disease with a high rate of mortality and no known reservoir other than human beings [30].

Although previous studies mention finding the DNA of *Leishmania* sp. and *Leishmania* (*L.*) *infantum* in the salivary glands of 10.1% of ticks, such as *R. sanguineus* collected from dogs, it was remarked that, despite the low percentage of parasitized ticks, there is probably a high parasitic load in each one of them [13]. On the other hand, this study found a high percentage of parasitized ticks in the collected animals.

In our study, *R. microplus* and *A. sabanerae* may were parasitized by ingesting contaminated blood of *P. tajacu* and *C. denticulata,* respectively. *P. tajacu* and *C. denticulata* are wild animals and could behave as reservoirs of *Leishmania*, so it is important to study *Leishmania* in the jungle niche. An interesting study mentions the laboratory’s infection of wild animals and the importance of knowing the sylvatic host and/or reservoirs in the control of leishmaniasis [31]. However, the role of *P. tajacu* and *C. denticulata* in the cycle of leishmaniasis is not documented. It is important to mention that ticks attached to animals or foliage can adhere to people who are dedicated to the hunting and raising of these animals, in addition to the people engaged in tourism, agricultural, and research activities. Therefore, they could represent a local and international public health risk.

A previous study has already shown the presence of *L.* (*L.*) *infantum* at the level of *R. sanguineus* in the blood of dogs, so a relationship of parasitism between the arthropod and host or reservoir is presumed, and this path can originate from the arthropod to the dog inoculating the parasite and/or the dog to the arthropod ingesting this parasite or in both directions [12]. Previous studies have shown the presence of DNA and RNA of *L. infantum* in ticks, fleas, and lice [20], collected mostly from dogs and, in a particular, case of a cat [14]. However, our study shows the molecular detection of *Leishmania* in *R. microplus* and *A. sabanerae* collected from a wild animal such as *Pecari tajacu* and *Chelonoidis denticulata* for the first time, thus expanding the distribution of the parasite’s ecoepidemiology.

Is important to note that the Madre de Dios region is the second after Cusco with the highest incidence of cutaneous leishmaniasis in Peru, and the Las Piedras district in the Madre de Dios Region is ranked as the second district after Tambopata with the highest rate of leishmaniasis [32], including leishmaniasis endemic villages such as Botijón and Chacra Gamitana in the Las Piedras district, where the ticks were collected.

This study did not identify the species of *Leishmania* parasite in *R. microplus* because the amount was very low to sequence the genome. Another limitation was not collecting more animal and tick samples in the jungle. It is necessary to establish complementary studies to determine the viability of the parasite in the arthropod in the presence in *P. tajacu* and *C. denticulata.*

## 5. Conclusions

In conclusion, *Leishmania* sp. was identified at high percentages in *Rhipicephalus microplus* and *Amblyomma sabanerae* from wild *Pecari tajacu* and *Chelonoidis denticulata* (between 34.1 and 5428.6) parasites per arthropod, and this could suggest that the ticks were parasitized by sucking the blood from the animals where they were collected. This is the first report about *Leishmania* parasites found in wild *Rhipicephalus microplus* and *Amblyomma sabanerae*, adding new information about the distribution and epidemiology of the parasite in sylvan areas.

## Figures and Tables

**Table 1:** Taxonomic determination and quantification of DNA of parasites per tick.

Detection Percentage of Studied Ticks Parasitized by *Leishmania* sp.	Gender	Taxonomic Classification of the Tick	Host	Concentration Range of DNA of *Leishmania* Per Tick (fg/µL)	Range Number of Parasites Per Tick
Total 22/23 (95.7%) Males (52.2%) Females (26.1%) Nymphs (17.4%)	12 Males (52.2%) 7 Females (30.4%) 4 Nymphs (17.4%)	*R. microplus*	*Pecari tajacu*	12–769	34.1–2197.1
**Detection percentage** **of studied** **ticks parasitized by *Leishmania* sp.**	**Gender**	**Taxonomic classification of the tick**	**Host**	**Concentration range of DNA of *Leishmania* per tick (fg/µL)**	**Range number of parasites per tick**
Total 9/10 (90%) Adult (100%)	10 Males (100%)	*A. sabanerae*	*Chelonoidis denticulata*	157–1900	448.6–5428.6

• Controls: 5ASK (*L. major*)13,216,468 fg/ µL, MHOM/PE/84/LC53 (*L. braziliensis*) 2,238,220 fg/µL.

## Data Availability

Not applicable.

## References

[1] Ginebra: Centro de Prensa, Organización Mundial de la Salud (2022). Leishmaniasis. https://www.who.int/es/news-room/fact-sheets/detail/leishmaniasis.

[2] Organización Panamericana de la Salud (2022). Leishmaniasis [Internet].

[3] Alvar J., Vélez I.D., Bern C., Herrero M., Desjeux P., Cano J., Jannin J., den Boer M. (2012). Leishmaniasis worldwide and global estimates of its incidence. PLoS ONE.

[4] Lopes E.G., Geraldo C.A., Marcili A., Silva R.D., Keid L.B., Oliveira T.M.F.D.S., Soares R.M. (2016). Performance of conventional PCRs based on primers directed to nuclear and mitochondrial genes for the detection and identification of *Leishmania* spp.. Rev. Inst. Med. Trop. Sao Paulo..

[5] Romero G.A., Guerra M.V., Paes M.G., Cupolillo E., Toaldo C.B., Macêdo V.O., Fernandes O. (2001). Sensitivity of the polymerase chain reaction for the diagnosis of cutaneous leishmaniasis due to *Leishmania* (*Viannia*) *guyanensis*. Acta Trop..

[6] Vergel C., Walker J., Saravia N.G. (2005). Amplification of human DNA by primers targeted to *Leishmania* kinetoplast DNA and post-genome considerations in the detection of parasites by a polymerase chain reaction. Am. J. Trop Med. Hyg..

[7] Coelho W.M.D., Bresciani K.D.S. (2013). Molecular and parasitological detection of *Leishmania* spp. in a dipteran of the species *Tabanus importunus*. Rev. Bras. Parasitol. Vet..

[8] Coutinho M.T.Z., Bueno L.L., Sterzik A., Fujiwara R.T., Botelho J.R., De Maria M., Genaro O., Linardi P.M. (2005). Participation of *Rhipicephalus sanguineus* (Acari: *Ixodidae*) in the epidemiology of canine visceral leishmaniasis. Vet. Parasitol..

[9] Trotta M., Nicetto M., Fogliazza A., Montarsi F., Caldin M., Furlanello T., Solano-Gallego L. (2012). Detection of *Leishmania infantum*, Babesia canis, and rickettsiae in ticks removed from dogs living in Italy. Ticks Tick Borne Dis..

[10] Dabaghmanesh T., Asgari Q., Moemenbellah-Fard M.D., Soltani A., Azizi K. (2016). Natural transovarial and transstadial transmission of *Leishmania infantum* by naïve Rhipicephalus sanguineus ticks blood feeding on an endemically infected dog in Shiraz, south of Iran. Trans. R. Soc. Trop. Med. Hyg..

[11] Chen Z., Liu Q., Liu J.-Q., Xu B.-L., Lv S., Xia S., Zhou X.-N. (2014). Tick-borne pathogens and associated co-infections in ticks collected from domestic animals in central China. Parasit Vectors.

[12] Gonçalves L.R., Filgueira K.D., Ahid S.M.M., Pereira J.S., Vale A.M.D., Machado R.Z., André M.R. (2014). Study on coinfecting vector-borne pathogens in dogs and ticks in Rio Grande do Norte, Brazil. Rev. Bras. Parasitol. Veterinária.

[13] Solano-Gallego L., Rossi L., Scroccaro A.M., Montarsi F., Caldin M., Furlanello T., Trotta M. (2012). Detection of *Leishmania infantum* DNA mainly in *Rhipicephalus sanguineus* male ticks removed from dogs living in endemic areas of canine leishmaniosis. Parasit Vectors.

[14] Salvatore D., Aureli S., Baldelli R., Di Francesco A., Tampieri M.P., Galuppi R. (2014). Molecular evidence of *Leishmania infantum* in *Ixodes ricinus* ticks from dogs and cats, in Italy. Vet. Ital..

[15] Nicolas L., Prina E., Lang T. (2002). Real-Time PCR for Detection and Quantitation of *Leishmania* in mouse tissues. J. Clin. Microbiol..

[16] Barros-Battesti D., Arzua M., Bechara H. (2006). Carrapatos de Importância Medico-Veterinaria da Região Neotropical: Um Guia Ilustrado Para Identificação de Espécies.

[17] Roche Diagnostics GmbH (2008). High Pure PCR Template Preparation Kit.

[18] Van Eys G.J., Schoone G.J., Kroon N.C., Ebeling S.B. (1992). Sequence analysis of small subunit ribosomal RNA genes and its use for detection and identification of *Leishmania* parasites. Mol. Biochem. Parasitol..

[19] Gomes L.I., Gonzaga F.M., De Morais-Teixeira E., de Souza-Lima B.S., Freire V.V., Rabello A. (2012). Validation of quantitative real-time PCR for the in vitro assessment of antileishmanial drug activity. Exp. Parasitol..

[20] de Morais R.C.S., da Cunha Gonçalves-de-Albuquerque S., e Silva R.P., Costa P.L., da Silva K.G., da Silva F.J., Brandão-Filho S.P., Dantas-Torres F., de Paiva-Cavalcanti M. (2013). Detection and quantification of *Leishmania braziliensis* in ectoparasites from dogs. Vet. Parasitol..

[21] Guerbouj S., Mkada Driss I., Guizani I. (2014). Molecular Tools for Understanding Eco-Epidemiology, Diversity and Pathogenesis of *Leishmania* Parasites. Leishmaniasis—Trends in Epidemiology, Diagnosis and Treatment.

[22] Wortmann G., Sweeney C., Houng H.S., Aronson N., Stiteler J., Jackson J., Ockenhouse C. (2001). Rapid diagnosis of leishmaniasis by fluorogenic polymerase chain reaction. Am. J. Trop. Med. Hyg..

[23] Viol M.A., Guerrero F.D., de Oliveira B.C.M., de Aquino M.C.C., Loiola S.H., de Melo G.D., de Souza Gomes A.H., Kanamura C.T., Garcia M.V., Andreotti R. (2016). Identification of *Leishmania* spp. promastigotes in the intestines, ovaries, and salivary glands of Rhipicephalus sanguineus actively infesting dogs. Parasitol Res. Parasitol. Res..

[24] Medeiros-Silva V., Gurgel-Gonçalves R., Nitz N., Morales L.E.D.A., Cruz L.M., Sobral I.G., Boité M.C., Ferreira E., Cupolillo E., Romero G.A.S. (2015). Successful isolation of *Leishmania infantum* from *Rhipicephalus sanguineus* sensu lato (Acari: *Ixodidae*) collected from naturally infected dogs. BMC Vet. Res..

[25] Magri A., Caffara M., Fioravanti M., Galuppi R. (2022). Detection of *Leishmania* sp. kDNA in questing Ixodes ricinus (Acari, *Ixodidae*) from the Emilia-Romagna Region in northeastern Italy. Parasitol. Res..

[26] Magri A., Galuppi R., Fioravanti M., Cafara M. (2022). Survey on the presence of *Leishmania* sp. in peridomestic rodents from the Emilia-Romagna Region (North-Eastern Italy) on the presence of *Leishmania* sp. in peridomestic rodents from the Emilia-Romagna Region (North-Eastern Italy). Vet. Res. Commun..

[27] Sgroi G., Iatta R., Veneziano V., Bezerra-Santos M., Lesiczka P., Hrazdislová K., Annoscia G., D’Alessio N., Golovchenko M., Rudenko N. (2021). Molecular survey on tick-borne pathogens and *Leishmania* infantum in red foxes (Vulpes vulpes) from southern Italy. Ticks Tick-Borne Dis..

[28] Cazan C., Ionica A., Matei I., D’ Amico G., Muñoz C., Berriatua E., Dumitrache M. (2020). Detection of *Leishmania* infantum DNA and antibodies against *Anaplasma* spp., *Borrelia burgdorferi* s.l. and *Ehrlichia canis* in a dog kennel in South-Central Romania. Acta Vet. Scand..

[29] Rojas-Jaimes J.E., Correa-Núñez G.H., Rojas-Palomino N., Cáceres-Rey O. (2017). Detección de *Leishmania* (*V*) *guyanensis* en ejemplares de *Rhipicephalus* (*Boophilus*) *microplus* (Acari: *Ixodidae*) recolectados en pecaríes de collar (*Pecari tajacu*). Biomédica.

[30] del Valle-Mendoza J., Rojas-Jaimes J., Vásquez-Achaya F., Aguilar-Luis M.A., Correa-Nuñez G., Silva-Caso W., Lescano A.G., Song X., Liu Q., Li D. (2018). Molecular identification of *Bartonella bacilliformis* in ticks collected from two species of wild mammals in Madre de Dios: Peru. BMC Res. Notes.

[31] Medina R. (1966). Leishmaniasis experimental en animales silvestres. Dermatol. Venez..

[32] Centro Nacional de Epidemiologia (2017). Prevención y Control de Enfermedades Casos de Leishmaniosis por años. Años 2000–2017*. Perú. http://www.dge.gob.pe/portal/docs/vigilancia/sala/2017/SE44/leishmaniosis.pdf.

